# Impacts of COVID-19 on reproductive health service provision, access, and utilization in Ethiopia: Results from a qualitative study with service users, providers, and stakeholders

**DOI:** 10.1371/journal.pgph.0001735

**Published:** 2023-03-23

**Authors:** Bekalu Mossie Chekol, Samuel Muluye, Grace Sheehy

**Affiliations:** 1 Ipas Ethiopia, Addis Ababa, Ethiopia; 2 Ipas, Chapel Hill, NC, United States of America; The Hospital for Sick Children, CANADA

## Abstract

Ethiopia has made significant improvements to sexual and reproductive health (SRH) in recent decades, yet the COVID-19 pandemic brought new challenges to SRH service delivery. In the early months of the pandemic, health facility and health management information system data showed reductions in SRH service utilization, however more evidence is needed on ongoing SRH impacts to inform policy and program decision-making. Our study aimed to assess the impacts of COVID-19 on SRH service utilization and access from the perspective of providers, clients, and stakeholders in Addis Ababa and Amhara Regional State. We collected data from May through July 2021 via six focus group discussions with health service providers, 50 in-depth interviews with SRH service clients and 15 key informant interviews with policy and programmatic stakeholders. All audio recordings were transcribed and translated from Amharic into English. Data were coded and analyzed for content and themes using Excel and NVivo 10. We found that COVID-19 and associated public health mitigation measures had consequences on SRH prioritization, funding, and service delivery. Efforts to halt the spread of COVID-19, such as gathering and transportation restrictions, also reduced access to SRH services. Fear of infection, costly and inaccessible transportation, commodity stockouts, and limited service availability all impeded access to and use of SRH services. For some women, this meant losing timely access to contraception and subsequently facing unwanted pregnancies. Providers experienced increased workloads, anxiety, and stigma as possible sources of infection. Our findings offer useful learnings for program and policy stakeholders aiming to meet SRH needs during the pandemic, and during times of crisis more broadly.

## Introduction

The COVID-19 pandemic has led to disruption, illness, and death around the world. As is common in times of crisis, the pandemic’s consequences have been experienced most acutely by those who already experience marginalization, exacerbating existing social and economic inequities [1]. In low-resource settings, the pandemic has contributed further strain to health systems already stretched thin, drawing resources away from often underfunded and neglected issues, such as sexual and reproductive health (SRH) [2–4]. COVID-19 has had both direct and indirect impacts on SRH outcomes. The risk of adverse outcomes, including maternal death, pre-eclampsia, preterm birth, and stillbirth, is elevated among pregnant individuals who contract the virus [5–7]. At the same time, efforts to mitigate the spread of COVID-19, such as travel restrictions and changes to health service delivery, have impacted the availability of SRH services, and subsequently SRH outcomes [8]. In a scoping review of research from across Africa, 78% of studies reported delayed or decreased access to maternal and child health services due to the pandemic [9]. Subsequently, preference for home birth increased, health facility visits and inpatient care decreased, and health system capacity was reduced, with added challenges faced by already vulnerable populations such as adolescents and displaced people [9, 10]. Marie Stopes International projected 1.3 million additional unintended pregnancies and 5,000 additional pregnancy-related deaths due to the pandemic in the 37 countries where they operate, including Ethiopia [11].

When COVID-19 was first detected in the east African country of Ethiopia, there was concern that the pandemic would threaten the strides the country has made in improving reproductive health over the past twenty years [12, 13]. Access to sexual and reproductive health care has improved considerably due to successful efforts to strengthen the country’s health system and develop supportive reproductive health policies [14]. From 2000 to 2018, per capita health expenditure increased from $5 USD to $27 USD [12, 15]. Access to essential health services has increased considerably: skilled birth attendance increased by 50%, receipt of antenatal care from a trained provider increased from 27% to 74% [12], use of modern contraceptive methods increased from 7% to 41% among married women of reproductive age, and maternal mortality decreased from 871 to 401 deaths per 100,000 live births [16, 17]. The abortion law was liberalized in 2005, expanding the legal grounds for abortion and the types of providers that can offer the service [18]. Despite this progress, gaps in access to SRH services and information persist, and the health system remains under resourced [12, 14].

Ethiopia’s first COVID-19 case was confirmed on March 13^th^, 2020. As of September 2022, there have been approximately 500,000 confirmed cases in the country, largely clustered in urban centers [12, 19, 20]. The government declared a federal emergency on April 8^th^, 2020, and multiple government bodies were set up to coordinate the COVID-19 response, as well as task forces and a surveillance and reporting system [21]. The government introduced numerous public health and social distancing mitigation measures, and short-term national and regional lockdowns. Some public hospitals were entirely diverted toward COVID-19 treatment and stopped providing other health services [12]. In the early months of the pandemic, health facility and health management information system data demonstrated significant reductions in inpatient and outpatient care in numerous parts of the country [22–24]. In Addis Ababa, significant decreases were noted in postnatal care, new family planning clients, and safe abortion care during the early months of the pandemic, with some trends reversing later in 2020 [22]. In southwest Ethiopia, survey research found significant reductions in use of antenatal care, facility births, and family planning in the early months of the pandemic, as well as increases in adolescent pregnancy and abortion [23], while qualitative data from pregnant women and providers uncovered barriers to accessing antenatal care, including COVID-19 mitigation measures, individual factors such as anxiety, and health facility barriers [24].

Much of the existing literature has demonstrated initial decreases in maternal and reproductive health service utilization early in the pandemic, with a rebound by mid-late 2020 [22–24]. Less evidence is available from the year following the acute stage of the public health emergency, particularly exploring the experiences and perspectives of service users, providers and key stakeholders who navigated the rapidly changing SRH service environment. In this study, we aimed to explore the impact of COVID-19 on sexual and reproductive health service delivery and utilization in two settings in Ethiopia: Addis Ababa City Administration and Amhara Regional State. Specifically, our study aimed to:

1. Examine how the health system was impacted by the pandemic, specifically with respect to SRH service delivery, funding and prioritization.
2. Identify how organizations, service providers and stakeholders adapted SRH programming and service provision.
3. Explore the impacts of COVID-19 and associated restrictions on women’s reproductive health service access and utilization.

## Methods

### Study setting

This paper draws on qualitative data collected by Ipas Ethiopia, a reproductive health non-profit organization focused on abortion and contraceptive services, to explore the impacts of COVID-19 on sexual and reproductive health (SRH) service delivery, accessibility, and utilization. We conducted this study in Addis Ababa Metropolitan area and Amhara Regional State. Addis Ababa Metropolitan area (hereafter referred to as Addis) is the capital of Ethiopia and comprises 10 administrative districts or sub-cities called kifle-ketemas and 116 woredas, which are the lowest administrative units. It is also the largest city in the country with an estimated population of 5.46 million [25]. The capital city covers 527 km^2^ of area. Most of the population in Addis is Orthodox Christian (82%), while 13% are Muslim [26]. Addis is home to a diverse mix of ethnic groups, with roughly half of its population belonging to the Amhara ethnic group, and the remainder split among Oromo, Gurage and Tigray [26]. One-third of Addis’s population is under the age of 18 [27]. Due to its size and density, the city experienced the country’s greatest concentration of COVID-19 infections, with 75% of confirmed cases in August 2020 [28].

Amhara Regional State (hereafter referred to as Amhara) is one of the country’s nine regions and is in northern Ethiopia. The state is 87% rural, and home to more than 22 million people, nearly one-quarter of Ethiopia’s total population [29]. In Amhara, there are 12 administrative zones, 3 metropolitan cities and 158 woredas and 46 towns. The regional state covers 154,709 km^2^ of area. Most of the state’s population are from the Amhara ethnic group, and the majority of the population is Orthodox Christian (83%), with a small Muslim minority [30]. Amhara’s population is youthful, with nearly 46% under the age of 18 [27].

These settings were selected due to their high COVID-19 case rates compared to other parts of the country, minimal security issues which could threaten research, and strong networks by Ipas in both settings which could facilitate data collection. We included female health service users, providers, and key stakeholders in both settings to obtain a broad understanding of the impacts of COVID-19 on SRH.

### Study design and sample

Our study team, all Ipas employees, collected data using multiple qualitative methods from May to July 2021. In brief, we conducted focus group discussions (FGDs) with 42 SRH care providers, in-depth interviews (IDIs) with 50 female health facility clients, and key informant interviews with 15 policy and program stakeholders (Table 1). We used purposive sampling and leveraged our networks as a non-profit working in SRH to reach out to health facilities (15 in Amhara and 10 in Addis) and relevant organizations to recruit participants. Potential participants were informed about the study and invited to participate by a study team member. Eight key informant interviews were conducted virtually, and all other data collection took place in person.

**Table 1:** Overview of data collection methods and sample.

Method	Sample	Number by region
**Focus group discussions (FGDs)**	42 providers (physicians, nurses, midwives, health officers) working in both public and private facilities	3 FGDs in Amhara 3 FGDs in Addis
**In-depth interviews (IDIs)**	50 female SRH service users	30 IDIs in Amhara 20 IDIs in Addis
**Key informant interviews (KIIs)**	9 representatives from CSOs and NGOs	9 KIIs in Addis
	6 state actors	4 KIIs in Addis 2 KIIs in Amhara

### Focus group discussions

We conducted six focus group discussions (FGDs) with 42 health service providers (three FGDs in Addis Ababa and three in Amhara). FGDs aimed to explore providers’ perspectives and experiences providing healthcare during the pandemic. FGDs were conducted in Amharic and led by trained local data collectors. FGDs were held in a private room in the health facilities where providers worked and covered topics including experiences providing health care during the pandemic, adaptations made to facility standards and supplies, and changes to individual provider practices due to COVID-19 (S1 Text). Participants worked in a range of roles providing SRH care, including as physicians, nurses, midwives, and health officers.

### In-depth interviews and key informant interviews

We conducted in-person in-depth interviews (IDIs) with 30 female health facility clients in Amhara and 20 in Addis, for a total of 50 IDIs. Our IDIs with female clients aimed to understand changes in SRH service access and use due to COVID-19. IDIs were conducted in Amharic and were led by trained interviewers. IDIs were held in a private room at the health facility where services were being sought. Women were recruited following receipt of family planning, abortion, post-abortion care, antenatal or postpartum care at designated facilities. Facility healthcare providers invited eligible women to participate in an in-depth interview about their experience accessing reproductive health services since the pandemic began. Interviews were conducted in a separate room to maintain privacy. The semi-structured IDI guide included topics such as experiences of and changes to cost, availability, and utilization of SRH services since the pandemic began (S2 Text).

Finally, we conducted 15 key informant interviews with representatives from community service organizations (CSOs) and non-governmental organizations (NGOs) working in sexual and reproductive health, as well as with policymakers and Ministry of Health staff. Our study team made appointments with key informants to explain the details of the interviews and conducted interviews at their place of work at a time convenient to them. We used a semi-structured interview guide for these interviews as well, which covered topics including the impacts of COVID-19 on government and program operations and funding, institutional COVID-19 responses and adaptations, and observed impacts of COVID-19 on communities’ sexual and reproductive health (S3 and S4 Texts).

### Ethics

We obtained ethical approval to conduct this study from the Ethiopian Public Health Association (EPHA) Ethical Review Board. We also sought relevant administrative approvals from health offices, health facilities and organizations targeted for interviews. All FGD and interview participants provided written consent to participate in the study prior to beginning data collection. We compensated providers and service users for their time. All interviews and focus groups were audio-recorded with participant consent, and the interviewers took notes of observations and visual cues that could not be captured by voice recorders. Data were stored in a secure online database. All online, hard copy and audio recordings were transferred to password protected laptops and backed up in the secure online database before being deleted from the source files. Audio recordings of interviews were labelled with unique identifiers and anonymized, and no names or identifying information appear in any study materials.

### Data analysis

Audio recordings from interviews and focus groups were transcribed and translated into English, when necessary, by a professional transcriber. Our study team first read through transcripts to familiarize ourselves with the content of the interviews. We analyzed interview and focus group data for content and themes using deductive and inductive analysis approaches [31]. Our analysis was guided by collaborative team discussions to identify key themes from our data. We coded data using NVivo, and then used Microsoft Excel to organize coded outputs by key themes and sub-themes. De-identified data are available in the S5 Text.

## Results

### Sample characteristics

Our sample included 50 women seeking health care services (hereafter referred to as health service clients), 42 health care providers, and 15 key informants representing NGOs, CSOs and governmental agencies (Table 2). Among the female clients, 30 were in Amhara and 20 in Addis Ababa. Service users ranged in age from 20 to 42, with an average age of 28. The majority (n = 33) were married, and most had less than a university education. Most women (n = 28) were seeking family planning services at the facility where they were recruited, while 9 were seeking abortion care.

**Table 2:** Sample characteristics of health service clients.

Female health service clients (n = 50)*	N	%
**Location**		
Addis Ababa	20	40
Amhara	30	60
**Age**		
20–24	11	27.5
25–29	11	27.5
30–34	11	27.5
35–39	5	12.5
40+	2	5
**Marital status**		
Single	5	23.5
Married	33	82.5
Divorced	2	5
**Service being sought at facility**		
Family planning	28	68.3
ANC	2	4.9
Abortion	9	22
Post-abortion care	2	4.9
**Highest education level**		
Primary or less	14	35
Secondary school	18	45
University	8	20
**Occupation**		
Unemployed	3	7.5
Housewife	8	20
Farming	3	7.5
Service industry	3	7.5
Health worker	2	5
Student	4	10
Teacher	4	10
Self-employed/ day laborer	6	15
Office worker (e.g. civil servant, bank)	7	17.5

*Note: Data missing on age, marital status, education and occupation for 1 participant, and all demographic data missing for 9 participants.

Among the 42 providers who participated in FGDs, 25 were in Amhara and 17 in Addis Ababa (Table 3). Slightly more providers were men (n = 24) than women (n = 18), and most participants worked as health officers (n = 20), while the remainder were nurses (n = 10), midwives (n = 11), or physicians (n = 1).

**Table 3:** Sample characteristics of providers.

Providers (n = 42)	N	%
**Location**		
Addis Ababa	17	40
Amhara	25	60
**Sex**		
Female	18	43
Male	24	57
**Occupation**		
Nurse	10	23.8
Midwife	11	26.2
Health officer	20	47.6
Physician	1	2.4

Finally, of our 15 key informants, 7 were female and 8 were male (Table 4). Key informants worked for NGOs (n = 4), CSOs (n = 5), state government (n = 2) and national government (n = 4). Most key informants (n = 13) were based in Addis, where most organizations are headquartered, and 2 were based in Amhara.

**Table 4:** Sample characteristics of key informants.

Key informants (n = 15)	N	%
**Location**		
Addis Ababa	13	87
Amhara	2	13
**Sex**		
Female	7	47
Male	8	53
**Organization type**		
Non-governmental organization	4	26.7
Civil society organization	5	33.3
Government (state level)	2	13.3
Government (national level)	4	26.7

### Key themes

We separated our key findings into three thematic domains with sub-themes where necessary: impacts of the pandemic on the Ethiopian health system; adaptations made to SRH programming, policy and service delivery; and impacts of the pandemic on sexual and reproductive health.

#### Impacts of the pandemic on Ethiopia’s health system

Providers, key informants, and service users alike described the significant impacts COVID-19 had on Ethiopia’s healthcare system, and subsequently on SRH services. Across the country, health services and resources were redirected toward the pandemic response; a lack of budgeting for emergency response meant that funds had to be redirected from SRH and other services when the pandemic hit. One key informant estimated that SRH funding had reduced by one-third.

Especially in the early months of the pandemic, health facility closures, travel and staffing restrictions, and lack of coordination between different levels of the health system led to the discontinuation of basic health system functions:

*The government response to the COVID-19 pandemic … had significant impact on SRH service provision. Most health facilities were closed, and transport service were restricted, health care providers were also restricted to go to the health facilities. The health system was collapsed, and coordination was so poor, the linkage between health facilities-health posts and health center and hospitals was severely affected.–Male CSO representative working in Addis*

Providers and key informants explained that the effects on antenatal and postnatal care have been particularly long lasting, extending beyond the early months of the pandemic into 2021.

Budget shortages hindered the availability of personal protective equipment (PPE), soap and water at healthcare facilities; stockouts of these supplies continued even as services began to resume. SRH commodities also faced shortages and stockouts due to loss of funding, reduced support from non-governmental organizations, delays in purchasing and distribution, and lack of coordination, all of which impeded service provision.

The pandemic also intersected with other ongoing crises in the country, such as poverty, conflict, and displacement. These crises made COVID-related disruptions more challenging to manage, further exacerbating commodity and service disruptions. At the same time, these crises made it harder to direct the attention and prioritization required to combat the spread of COVID-19:

*We have shortage of drugs and supplies due to COVID-19 disruption and due to large number of displaced people (from Benishangul) seeking the service. In my Woreda [district], you can’t get family planning commodities in most of the health facilities, except the injectable.–Female MCH worker in Amhara*

*There are very poor people who can’t afford to purchase face mask which the health facilities start to support them. Furthermore, there are civilian deaths due to political crisis. There is also war and the issue of COVID and health is forgotten- it lacks attention.–Male MCH worker in Amhara*

Some providers stopped providing SRH services and shifted toward COVID-related services during the early stages of the pandemic. Most providers described facing increased and changing service demands, including covering shifts for colleagues who were infected with COVID-19:

*Additional tasks associated with COVID-19 vaccination is becoming another burden for health care providers and it affects SRH service provision. COVID-19 increased workload among us, we should stretch ourselves to cover the work of staffs who died of and infected with COVID-19 virus, there was no immediate replacement for these providers–Midwife working in Addis*

In addition to physical health impacts, the pandemic also impacted the mental and social well-being of providers. Some providers explained the fear and anxiety they felt about contracting COVID-19, and many described how they had faced stigma within their facilities and their communities as they were feared as a possible source of COVID-19 infection:

*Health providers’ social interaction and engagement was severely affected by COVID-19 pandemic. I personally was unable to get rental house because of the community stigma. They perceived me as the carrier of COVID.–Female nurse working in Addis*

#### Adaptations made to SRH policy and programming, and service delivery due to COVID-19

A considerable focus of the interviews and focus group discussions was on the adaptations made to SRH programming, policy, and service delivery due to COVID-19. This section divides this theme into two sub-themes: shifts in funding and prioritization of SRH; and modifications to SRH programming and service delivery.

*Shifts in funding and prioritization of SRH.* As the pandemic first hit the country in early 2020, the Ethiopian government, healthcare providers, and SRH organizations all made rapid adaptations to policy and funding. While considerable attention shifted to treating and preventing COVID-19 infections, at times at the expense of other health services, important efforts were made to maintain and promote SRH service delivery, including through integration with COVID-19 efforts. Key informants described how they advocated for the integration of SRH activities within COVID-19 prevention and treatment activities:

*We motivated and engaged the health work force to integrate SRH activities with COVID-19 prevention and treatment activities–Male CSO representative working in reproductive health in Addis*

*We did not recommend COVID-19 related interventions as a stand-alone initiative rather it should be integrated with other health and non-health programming–Male NGO representative working in reproductive health in Addis*

To ensure prioritization of SRH service provision, task forces were created, service provision harmonization and essential health service guidelines were developed, and health extension workers were tasked with maintaining service delivery beyond their normal scope of work. Some NGOs also deployed staff to provide technical support to SRH programs across health system levels. However, understanding and implementation of newly produced guidelines varied:

*Many SRH guidelines and documents have been prepared in the context of the COVID-19 pandemic. … Essential health service guidelines were developed to maintain SRH services. After the implementation of essential service guidelines, SRH became among the priority services in the context of the COVID-19 pandemic and SRH service provision issues started to diminish, although providers did not have uniform understanding on the guideline–Male CSO representative working in Addis*

Much funding and effort were directed toward purchasing and distributing PPE for health facilities. Key informants used their networks to help mobilize PPE so that providers in public health facilities could continue offering SRH services:

*We tried to develop initiatives to generate fund for PPE support. As a result, we were able to support the health system with face masks, sanitizer, and soap. We also engaged in distributing PPE materials from high resource districts to low resource districts.–Male policymaker working in Amhara*

However, a lack of emergency preparedness budgeting meant that considerable funds were shifted away from SRH and other health services to direct funding toward the COVID-19 response, contributing to stockouts of SRH commodities, among other issues:

*There was no pre-planned budget for emergency responses such as COVID-19 and most of the COVID-19 response activities utilized up to 30% of SRH and other health program budget.–Male NGO representative working in Addis*

*There was shortage of [medication abortion] drugs in our health facility. We refer the client to private pharmacy to purchase the drugs.–Female HIV officer working in Amhara*

Ultimately, the pandemic provided both opportunities and challenges when it came to funding SRH services and operations:

*We replanned our SRH project budget to purchase PPE materials since we did not have budget for COVID-19 prevention. Funders have also started to shift their budget towards COVID-19 prevention. COVID-19 pandemic has also become an opportunity for funding; some donors had supported the COVID-19 relief and response strategies–Male NGO representative working in in Addis*

*Modifications to SRH programming and service delivery:* Health facilities across Addis and Amhara implemented public health measures, such as masking and social distancing, to reduce the spread of COVID-19. This included modified SRH service schedules to accommodate the need for COVID-19 services and reduce the frequency of potential COVID-19 exposure at health facilities:

*Health facilities were providing SRH services with no mask–no service rule, they also adjusted seating service provision arrangements to prevent COVID-19 infection. Service appointments were also extended, ART drug appointment extended to 6 months, ANC attendance changed from 4 visits. We extended population catchment areas for COVID-19 prevention and management, and it was our day-to-day priority tasks–Female policymaker working in Addis*

*We reduced the frequency of visiting health facility for lactating and pregnant mothers to minimize their risk of COVID-19 infection–Female health officer working in Addis*

However, some service users described their frustration by a lack of enforcement of public health measures, a lack of hygienic supplies, and crowding in health facilities:

*The health facilities enforced use of face mask at the entrance. There is hand washing facility but it’s not functional and has no soap and water. There is no controlling system for face mask use in health facility– 22-year-old single student in Amhara*

SRH programs and services adopted virtual service delivery models where possible. Some providers began offering virtual support and counseling, including for provision of drugs such as antiretrovirals and medication abortion. NGO representatives described how their organizations shifted to virtual means of reaching clients and patients, including using social media and online platforms to connect with communities, staff, and providers:

*We started to develop online platforms to reach and connect with trained providers. We also have started to initiate and introduce telemedicine in some big cities. Some staffs work at home using telephone and online communication means. In person trainings has been reduced from our plan and we have started to conduct only theoretical trainings through online system–Male NGO representative working in Addis*

However, poor connectivity posed a challenge to virtual models of work: *We were sometimes challenged by connectivity problem or when the internet become down or unreliable–Male NGO representative working in Addis*.

In addition to virtual offerings, NGOs also adapted formerly in-person programs and activities to comply with public health guidance while still reaching communities to increase demand for SRH services and provide education about COVID-19. In-person outreach was appreciated by many service users.

*We had difficulty to create demand for SRH services in the community, since gathering of people was restricted, but we used our promoters to create demand for service through home-to-home visits by applying COVID-19 infection prevention strategies–Male CSO representative working in Addis*

*Most women are not visiting health facilities in fear of COVID-19 infection. The SRH information and services should be provided door to door through health extension workers/ community health workers.– 25-year-old married self-employed woman in Addis*

#### Impacts of COVID-19 and associated restrictions on SRH service access and utilization

The pandemic and associated mitigation measures had varied impacts on SRH access, use, and outcomes. We have divided this domain into three sub-themes: COVID-19 impacted contraceptive access, with consequences for women’s health; public health measures at facilities were appreciated but prohibitive; and COVID-19 exacerbated pre-existing barriers to SRH access.

*COVID-19 impacted access to contraception, with consequences for women’s health.* Early in the pandemic, the availability of SRH services decreased dramatically as provision of many services were discontinued. When services resumed in mid-2020, fears about COVID-19 exposure in facility settings continued to impact service utilization. Key informants described increases in home deliveries, unwanted pregnancies, and unsafe abortion due to reduced service availability and use. This sentiment was echoed by many female service users who described their own reticence to visit health facilities, particularly for family planning services:

*I delayed removal of the [contraceptive] implant; I couldn’t come on time because of fear of COVID-19 infection. At the start of the pandemic, everything was scary and affected our movements. I stayed at home two months when the pandemic started and delayed the removal.– 42-year-old married self-employed woman in Addis*

For many women in our study, regular access to contraception became more challenging during the pandemic. Stockouts of preferred contraceptive methods and concerns about COVID-19 exposure in facilities impacted their ability to maintain consistent contraceptive use and prevent unwanted pregnancies. Several abortion clients in our study became pregnant because they had struggled to access family planning during the pandemic:

*I come to terminate my unintended pregnancy … I did not use family planning during COVID time. I was afraid to go to the health facility because of COVID … Before COVID I took what I need. I took depo [Depo-Provera] for three months. It was available. But during COVID I could not get depo in the health post and the provider gave me oral pills for one month. I forgot to take pills and now I faced unwanted pregnancy– 22-year-old single student in Amhara*

This was particularly challenging for some for whom the need for contraception increased during lockdown. As one participant, as 23-year-old married teacher, described: *During COVID*, *the demand for sexual intercourse from my husband increased*, *and I ended up with an unplanned pregnancy*.

Some providers told us that they counselled women to shift to longer-acting family planning methods, such as implants and IUDs, to ensure protection against pregnancy could be sustained with fewer in-person clinic visits. One service user described switching from the injectable to the implant due to challenges reaching the health facility:

*Previously I used the injectable method but now I use the FP for three years [implant]*. *This is because I have difficulty to get transport*.– *23-year-old married farmer in Amhara*

However, the push to long-acting methods was not favored by all participants—as one service user in Addis described:

*Some providers are pushing clients to prefer a specific method, especially LARC [long-acting reversible contraceptive] methods and on the other hand, some women have misconceptions towards LARC methods. So, there must be a strategy to reduce providers’ biasness and alleviate clients’ misconceptions.– 26-year-old married public servant in Addis*

*Public health measures at facilities were often appreciated, but prohibitive.* Despite changes to service delivery due to the pandemic, many service users had positive experiences obtaining SRH care. Many stated that access to SRH services had not significantly changed for them since the pandemic began, except for longer waiting times and new public health measures in the facilities. Providers described how some service users complained about having to wear a face mask to receive services:

*No mask no service rule and guidance were one of the complaints of service users. Clients do not want to use face mask–Male MCH worker in Amhara*

However, in our in-depth interviews, most service users expressed minimal issues with complying with masking policies, apart from the costs of having to purchase a mask. For some, the cost of a face mask was prohibitive and kept them from receiving needed SRH care, or required they share with others to access the health facility:

*During COVID*, *providers enforced use of face masks*. *It’s difficult to get a face mask if I have no money*.– *23-year-old married farmer in Amhara*

*Women give their face mask to other people after they leave the health facility.– 27-year-old married teacher in Amhara*

Similarly, efforts to maintain social distancing in health facilities were generally appreciated by service users, however some complained about COVID-19 clients not maintaining social distancing, and many described long wait times in clinics due to COVID mitigation measures:

*There were many service seekers around the registration (card) room. Most people do not keep their distance from others. The health provider did not provide the service timely-I had to wait long time to get the service.– 30-year-old married unemployed woman in Addis*

*The health care providers are taking protective measures to prevent COVID-19 infection, so they took a little longer time to serve a client. As a result, we wait a bit longer time to get the service.– 33-year-old married health worker in Addis*

*COVID-19 exacerbated pre-existing geographic and social barriers to SRH access.* For some women in our study, geographic barriers to access intersected with pandemic-mitigation measures (and other barriers, such as lack of partner support) to further render SRH services inaccessible. Several service users described the implications of these intersecting challenges on their reproductive health outcomes, including dealing with unwanted pregnancies:

*I do not have time to visit health facility. The health facility is far from my residence … My boyfriend is not willing to visit this health facility. I would take FP service if the health facility is near and my partner encouraged me. Because of distance, lack of information and lack of willingness from my partner I [was] exposed for unwanted pregnancy. -22-year-old unmarried student in Amhara*

To prevent the spread of COVID-19 on public transportation, restrictions were placed on the number of people permitted in public transportation vehicles at a given time. This increased costs and reduced the availability of transportation services, which many service users described as a major barrier to accessing health facilities:

*Although the facility is not far from my home, there is transportation problem here in Addis Ababa, especially in this difficult time. I had to wait a long time to get a taxi and visit this facility.– 33-year-old married health worker in Addis*

*Due to fear of COVID and transport problems I stopped using family planning. During COVID I faced an unwanted pregnancy, and I delivered a child without my plan. COVID affected my family planning use and had great impact on my life. -23-year-old married farmer in Amhara*

Rural-urban disparities were evident in access to both SRH and COVID-19 information and services, and these disparities were further aggravated due to the pandemic. While many participants in Addis described the health facility as convenient to reach, participants based in more rural Amhara explained how their already limited access to healthcare was further strained or entirely impeded when transportation options diminished, and health facilities closed:

*Health posts need to be opened. Usually, health posts are closed. I need services near to my home. I need COVID vaccine like that of urban people.– 35-year-old married farmer in Amhara*

*The urban community have information to prevent COVID and use SRH services. But rural community do not have the chance to get information … Sometimes we may not get providers on the time when we need, providers should be available every time.– 32-year-old married teacher in Amhara*

## Discussion

This paper provides evidence on the impacts of COVID-19 on sexual and reproductive health service delivery, access, and utilization in Ethiopia from the perspective of female service users, healthcare providers, and policy and programmatic stakeholders. We find considerable impacts of COVID-19 and associated mitigation measures on the delivery of SRH care, as funding and prioritization shifted to addressing COVID-19. Fear of infection, costly and inaccessible transportation, and limited service availability all impeded access to and use of SRH services with consequences for women’s health, including unwanted pregnancy. Our findings offer useful learnings for program and policy stakeholders aiming to meet SRH needs during the pandemic, and in times of crisis more broadly.

We found that the Ethiopian health system was significantly affected in the early months of the pandemic, as were health systems in many countries [1]. Service provision shifted dramatically to accommodate the need for COVID-19 treatment and prevention efforts, at times at the expense of essential SRH services. This finding is reflected in other research as well; a scoping review of studies from across Africa found disruptions in maternal and child health service provision were a common theme in countries including South Africa and Kenya as well [9]. Declines in utilization of sexual and reproductive health services in the early months of the pandemic have been documented in numerous countries, such as Nigeria, South Africa, and Pakistan [32, 33], as well as prior research in Ethiopia [12, 24]. As in our study, fear of COVID-19 infection was a common factor impeding utilization of SRH services across countries, often extending beyond the initial months of the pandemic and continuing to impede access once service provision resumed to pre-pandemic levels [21, 33]. In rural Ethiopia, uptake of antenatal care was hindered by anxiety related to the pandemic as well as public health mitigation measures and poor service quality [24]. We also found frustrations among service users about crowding and lack of enforcement of social distancing in facilities; improving quality of care is imperative both for reducing the risk of COVID-19 and improving SRH outcomes.

In line with other research in Ethiopia, stakeholders in our study noted that utilization of many SRH services recovered to pre-pandemic levels by mid-2020. However, for many service users, even a temporary decrease in access to SRH care had long-lasting and significant consequences, such as unwanted pregnancies. We found that the pandemic impacted many women’s ability to continue accessing their preferred methods of contraception; a finding echoed in other research in Ethiopia [12]. Stockouts of reproductive commodities were also documented as a major barrier to access in Pakistan and Nigeria [33, 34]. Longitudinal research from Burkina Faso and Kenya found that women who switched contraceptive methods were more likely to adopt more effective, long-acting methods of contraception [35]. Similarly, research in Senegal found a statistically significant shift from short-term to long-acting contraceptive methods, like IUDs and implants, during the pandemic [36]. This evidence echoes our finding that women often switched to longer-acting methods of contraception, and our data suggests this was often at the recommendation of providers to reduce the frequency of facility visits. However, given the extensive evidence on coercive practices surrounding LARCs [37, 38], more attention is needed on the practice of recommending LARCs during times of crisis.

Prior to COVID-19, urban-rural disparities in access to SRH services in Ethiopia were well-documented [39, 40]. We found that geographic barriers to access became more pronounced during the pandemic, particularly due to limited and costly transportation options, and inequities in availability of both COVID-19 and SRH services. Several studies in Nigeria similarly found transportation was a significant barrier to SRH service access and delivery [32, 34]. Service users in our study recommended more community outreach efforts to improve access to SRH services and information: provision of transportation to health facilities, door-to-door outreach and increasing the availability of services at health posts or via mobile clinics are all measures that could improve access to SRH services, especially in rural parts of the country. Service users also described the financial burden of purchasing masks to wear at facilities, which has been found in other settings as well [41]. Providing masks at low or no cost could be an important intervention for improving utilization of facility-based health services when masks are required for receipt of services.

Despite the challenges posed by COVID-19 to the Ethiopian health care system, participants in our study described myriad efforts made by government, NGOs, and providers to meet the health needs of the population through the most trying phases of the pandemic. That many SRH services resumed within months of the start of the pandemic speaks to the resilience of the Ethiopian health care system and the considerable work done by decision-makers and stakeholders. Efforts taken to maintain continuity of service delivery in Ethiopia mirror those taken in various low- and middle-income country contexts [42]. Other countries in the region, such as Kenya, Uganda, Mozambique and Zimbabwe similarly developed SRH guidelines for navigating the pandemic while preserving SRH service delivery [43]. However, participants in our study spoke to variable implementation of SRH guidelines during the pandemic, suggesting a need for more monitoring and supervision to guide the implementation process. Many participants spoke of advocacy efforts to integrate SRH into the COVID-19 response; flexible funding is useful for agile budgeting in this regard.

Several lessons can be gleaned from Ethiopia’s experience that may be useful for future COVID-19 or health crisis mitigation efforts. First, integrating SRH interventions within emergency response measures is important for maintaining consistent SRH service availability, and ensuring scarce resources are used effectively. Maintaining the supply of reproductive health commodities, and personal protective equipment, is imperative to ensure service delivery can continue in times of health system strain. Multisectoral strategies and coordination across different levels of the health system could help mitigate the spread and severity of disease and minimize impacts on other health services. Widespread provision of COVID-19 testing and treatment services should also be prioritized, and efforts made to address vaccine resistance and hesitancy. Budgeting for research and evaluation activities will help ensure that experiences, lessons, and recommendations can be captured and shared, while strengthening national disease surveillance systems will facilitate the tracking of cases to inform response efforts. Campaigns to share information about COVID-19 and other health issues should be tailored toward different audiences and use a wide range of platforms, including local media, social media, and messaging apps.

Our study is not without limitations. Our in-depth interviews were conducted with women receiving SRH services at health facilities; thus, we are unable to include the perspectives of those who were unable or unwilling to receive facility-based care and the barriers they faced. Similarly, our interviews took place within health facilities, which could have affected the information service users were willing to share, including less willingness to describe negative aspects of their experience [44, 45]. Interviews and focus group discussions were conducted in Amharic and translated to English, and it is possible some nuance was lost in the translation process. Our study is strengthened by the inclusion of multiple perspectives from both urban and rural parts of the country. We also used data collected in mid-2021, which is more recent than many published studies which examined the impacts of the pandemic in its early months. Thus, we were able to capture some longer-term implications, such as the consequences of diminished access to contraceptives on women’s reproductive health.

In conclusion, our findings demonstrate the major impacts COVID-19 has had on Ethiopia’s health system, as well as on access to and use of SRH services, in both Addis and Amhara. Transportation costs, inaccessible health services, and fear of infection all impeded timely and regular access to SRH services, including contraception. SRH service delivery efforts must adapt in times of crisis, such as via community outreach and telemedicine, to ensure continuity of care. Continuing to prioritize SRH service delivery in times of crisis is of the utmost importance, particularly for populations who already face barriers to accessing health services.

## Supporting information

S1 Text Focus group discussion guide. (DOCX)

S2 Text In-depth interview guide health service clients. (DOCX)

S3 Text In-depth interview guide NGO and CSO representatives. (DOCX)

S4 Text In-depth interview guide policymakers. (DOCX)

S5 Text Table of de-identified transcript data. (DOCX)
