# Supplementary material for: Impacts of COVID-19 on reproductive health service provision, access, and utilization in Ethiopia: Results from a qualitative study with service users, providers, and stakeholders
Source: PLOS Glob Public Health. 2023 Mar 23;3(3):e0001735. doi: 10.1371/journal.pgph.0001735 (PMC10035746; doi:10.1371/journal.pgph.0001735)
Supplement: S1 Text — (DOCX) [file pgph.0001735.s001.docx]

**Focus Group Discussion Guide: Service Providers**

**Information on this study**

We are trying to understand the impacts of the COVID-19 pandemic on the availability, access, and utilization of reproductive health services in Amhara Regional state and Addis Ababa City Administration. The study explores these impacts through information gathered from various sources, and also using perspectives of several diverse key informants. We will use this information to guide policy debates and advocacy messaging towards prioritization of SRHR even as governments respond to COVID-19 in Ethiopia.

I want to thank you for taking the time to meet with me today. We want to speak to you because your organization is involved in sexual and reproductive health in Ethiopia, and we value your knowledge and experience. If you have any questions for me during our conversations, please ask.

**Discussion guide**

1. Have there been any changes to service delivery guidelines from the MOH since the COVID 19 pandemic? If so, what changes?
2. Have there been any changes to the service delivery guidelines of your organization? If so, what changes?
3. Have you chosen to make any other changes to the way that you provide services or refer patients because of the COVID 19 crisis? If so, what changes?
4. Have there been any other changes in your organization that affect service providers, or service provision that are due to something other than the COVID 19 pandemic? If so, what changes?
5. Has the availability of commodities, like contraceptive methods, and drugs, like ARVs, changed in any way since COVID pandemic began? To what degree are these changes related to the COVID 19?
6. Does your organization provide or refer to POST-ABORTION CARE services? Has this component of your work changed at all since the COVID-19 crisis began?
7. Does the COVID 19 pandemic have an impact on health care service provision and access outside of SRH? If so, how?
8. What do you expect to be the biggest impacts of the current crisis (COVID 19) in this country? Do you expect certain geographic areas or populations to be affected the most? Which ones?
9. What, if at all, have you heard women (and young people) say, about changes that they have experienced seeking and receiving SRH services? *Probe on access, cost, availability, quality; experiences at public vs. private facilities*
10. *Does your facility provide services for young people? Have there been any youth friendly services provided?*
11. *Have you received any support from the government (MOH) to facilitate provision of SRH services?*
12. *What other support do you require from MoH to ensure continued service provision during this period?*

**Wrap-up**

Is there anything else that you would like to add or discuss here that you think would be relevant to the issue?

Do you have any questions or concerns?

Thank you very much for your time.

***Use probes when needed:**

- *Would you give me an example?*
- *Can you elaborate on that idea?*
- *Would you explain that further?*
- *I’m not sure I understand what you are saying.*
- *Is there anything else you’d like to share about that?*
