# Supplementary material for: Impacts of COVID-19 on reproductive health service provision, access, and utilization in Ethiopia: Results from a qualitative study with service users, providers, and stakeholders
Source: PLOS Glob Public Health. 2023 Mar 23;3(3):e0001735. doi: 10.1371/journal.pgph.0001735 (PMC10035746; doi:10.1371/journal.pgph.0001735)
Supplement: S2 Text — (DOCX) [file pgph.0001735.s002.docx]

**In-Depth Interview Guide: Client interviews**

**Information on this study**

We are trying to understand the impacts of the COVID-19 pandemic on the availability, access, and utilization of reproductive health services in Amhara Regional state and Addis Ababa City Administration. The study explores these impacts through information gathered from various sources, and also using perspectives of several diverse key informants. We will use this information to guide policy debates and advocacy messaging towards prioritization of SRHR even as governments respond to COVID-19 in Ethiopia.

I want to thank you for taking the time to meet with me today. You were selected for this interview today because you sought X service on X date

**Participant’s socio demographic characteristics**

| County |  |
| --- | --- |
| Facility |  |
| Age |  |
| Marital status |  |
| Service (s) sought |  |
| Highest education level completed |  |
| Occupation |  |

**Interview questions**

1. Could you please tell me how you ended up in this facility?

***Probe:***

*For what services*

*Was this the first time you were seeking this service or you have been here before COVID 19 pandemic?*

*Any differences in services/service provision from before?*

1. Considering when you wanted to visit the health facility, could you talk about any challenges you may have faced.

***Probe:***

*Lack of transport/Fare*

*Distance too long*

*COVID 19 Lock down/Curfew/restrictions in movement*

*Lack of time*

*Information on health facilities to visit*

*Partner or parent did not want me to come to the facility*

1. Could you tell us briefly how experiencing the above challenge affected you or your health?
2. Considering the challenge above, could you talk about how you coped with the situation?
3. In your opinion, what was the experience like when you came to the facility for care (before and during the COVID 19 pandemic)?

***Probe:***

*Did you get the service you wanted?*

*Were you satisfied with the service?*

*What did you like most about your experience receiving care? waiting time, privacy, friendliness of providers, health facility cleanliness etc.*

1. In the process of seeking care, what measures have you taken to reduce the risk of contracting COVID 19?

*Probe:*

*Any challenges?*

1. What have you observed as the steps taken by the health facilities to reduce the risk of COVID 19 while seeking care?

*Probe;*

*Do you have any difficulties complying with these measures?*

1. Could you tell us if any, how COVID 19 pandemic has affected your choice of SRH services? Please explain
2. In your opinion, what do you think could be done differently to ease access to:
3. SRH information during COVID 19
4. SRH services during COVID 19

**Wrap-up**

Is there anything else that you would like to add or discuss here that you think would be relevant to the issue?

Do you have any questions or concerns?

Thank you very much for your time.
