# Supplementary material for: Impacts of COVID-19 on reproductive health service provision, access, and utilization in Ethiopia: Results from a qualitative study with service users, providers, and stakeholders
Source: PLOS Glob Public Health. 2023 Mar 23;3(3):e0001735. doi: 10.1371/journal.pgph.0001735 (PMC10035746; doi:10.1371/journal.pgph.0001735)
Supplement: S3 Text — (DOCX) [file pgph.0001735.s003.docx]

**In-Depth Interview Guide: NGOs and CSOs representatives**

**Information on this study**

We are trying to understand the impacts of the COVID-19 pandemic on the availability, access, and utilization of reproductive health services in Amhara Regional state and Addis Ababa City Administration. The study explores these impacts through information gathered from various sources, and also using perspectives of several diverse key informants. We will use this information to guide policy debates and advocacy messaging towards prioritization of SRHR even as governments respond to COVID-19 in Ethiopia.

I want to thank you for taking the time to respond to this questionnaire today. We want to speak to you because of your involvement and work in sexual and reproductive health services at this health facility, and we value your knowledge and experience regarding this subject. If you have any questions for me during our conversations, please ask

**Interview questions**

1. What are your organization’s core area of work / priorities?
2. What is your organization doing to ensure that SRHR remains a priority during the COVID-19 pandemic?
3. What steps have you taken to support governments to address the COVID 19 pandemic?
4. What is the nature of support you are giving non state actors to address the COVID 19 pandemic?
5. How, if at all, has the COVID 19 pandemic directly or indirectly affected your work plan/activities and what have you done to mitigate/ cop?
   1. Has it affected your own individual work responsibilities? If so, how?
   2. Has your organization changed service provision or service delivery protocols because of COVID-19?
   3. Have you begun or ended certain services because of the COVID-19 pandemic?
   4. Workload, working hours, closing/ stopping to offer some services: *what other reasons*
6. Can you describe how your organization has adjusted to still deliver services during the COVID 19 crisis?
7. Have you or your organization come across/handled any human rights violations during the COVID 19 period? Which ones?
   1. What have you/your organization done to address these violations?
   2. Is there an existing mechanism to address violations of the right to health during this pandemic?
   3. Is the judiciary in your country well prepared to deal with any human rights violations happening during the COVID-19 pandemic? Explain your answer…………………
8. How has your funding been affected by the COVID 19 pandemic? …
   1. *When did this occur?*
   2. *Was this related to COVID-19? Tell us more?*
   3. *How has the change in funding affected your operations/ services?*
   4. *What proportion of your SRH funding has been affected by COVID-19 pandemic? [Only for organizations that are SRHR funded]*
   5. *What strategies have you put in place?*
9. How has the COVID 19 pandemic has impacted civil society and public discussions on SRHR?
   1. *Teenage pregnancy*
   2. *Safe abortion*
   3. *Contraception*
   4. *SGBV*
   5. *HIV/AIDS*
   6. *Child marriage*
   7. *Mental health*

*Any other area*

1. How, if at all, has the National government response to COVID-19 pandemic affected SRH service provision?
   1. *Do you think SRH has been adequately addressed during the COVID-19 pandemic?*
   2. *What measures have been put in place by the government to ensure accountability?*
   3. *Changes priority on funding for SRH?*
   4. *Instituted new policies and programs and service provision guidelines (abortion, SRH, HIV, other global health issues)?*
   5. *Changed how they communicate about SRH/abortion with your organization, partners or to the public [online platforms, IEC materials, telemedicine, WhatsApp, chatbot.*
   6. *To what extent is the government involving local communities in communicating and implementing COVID19 response directives?*
2. What do you recommend for your government to do to immediately improve the situation?
3. *What do you want the donor community to do to support the response to COVID-19 pandemic?*
4. Can you talk about the effects that the COVID 19 pandemic has on the service providers and health facilities that you work with?
   1. *How has service provision changed?*
   2. *Are certain services no longer offered?*
   3. *Staffing cuts?*
   4. *Clinic closures? Or changes to clinic hours?*
   5. *Have providers changed their behaviors or practices? Have they expressed concern or confusion?*
5. What do you expect to be the effects of the COVID-19 pandemic on SRH in the country?
   1. *Unintended pregnancy including teenage pregnancy*
   2. *unsafe abortion*
   3. *SGBV*
   4. *HIV/AIDS*
   5. *Child marriage*

*Do you expect certain geographic areas or populations to be affected the most?*

*Which ones?*

*Why?*

1. *Are there any reported increase in incidences of*
   1. *GBV Yes/No*
   2. *maternal deaths Yes/No*
   3. *unsafe abortions Yes/No*
   4. *Teenage pregnancy Yes/No*
   5. *FGM Yes/ No*
   6. *Others (specify)………………………………………..*
2. *Specifically, what action(s) has your government taken to address these incidences?*
3. *GBV…………………………………*
4. *Maternal deaths……………………………*
5. *Unsafe abortions……………………………*
6. *Teenage pregnancy ………………………..*
7. *FGM ……………………………..*
8. *What would you want to see governments, CSOs and NGOs do going forward?*

***Wrap-up***

1. Is there anything else that you would like to add or discuss here that you think would be relevant to our understanding of the impacts of COVID 19 crisis?
2. Do you have any questions or concerns?

Thank you very much for your time.

***Use probes when needed:**

1. *Would you give me an example?*
2. *Can you elaborate on that idea?*
3. *Would you explain that further?*
4. *I’m not sure I understand what you are saying.*
5. *Is there anything else you’d like to share about that?*
