# Supplementary material for: Impacts of COVID-19 on reproductive health service provision, access, and utilization in Ethiopia: Results from a qualitative study with service users, providers, and stakeholders
Source: PLOS Glob Public Health. 2023 Mar 23;3(3):e0001735. doi: 10.1371/journal.pgph.0001735 (PMC10035746; doi:10.1371/journal.pgph.0001735)
Supplement: S4 Text — (DOCX) [file pgph.0001735.s004.docx]

**In-depth interview guide: Policy makers/State Actors**

**Information on this study**

We are trying to understand the impacts of the COVID-19 pandemic on the availability, access, and utilization of reproductive health services in Amhara Regional state and Addis Ababa City Administration. The study explores these impacts through information gathered from various sources, and also using perspectives of several diverse key informants. We will use this information to guide policy debates and advocacy messaging towards prioritization of SRHR even as governments respond to COVID-19 in Ethiopia.

I want to thank you for taking the time to respond to this survey questionnaire today. We want to speak to you because of your involvement and work in sexual and reproductive health services in this country, and we value your knowledge and experience regarding this subject. If you have any questions for me during our conversations, please ask

**Interview questions**

1. How has COVID-19 affected government operations?
2. What was the government responses to COVID-19? *(what policies did the government put in place)*. Specifically, what action(s) has the government taken to address the following incidences?
   1. *Gender Based Violence (GBV)…………………………………*
   2. *Maternal deaths……………………………*
   3. *Unsafe abortions……………………………*
   4. *Teenage pregnancy ………………………..*
   5. *Female genital mutilation (FGM) ……………………………..*
3. To what extent is the government involving local communities in communicating and implementing COVID19 response directives?
4. What steps has the government taken to provide public information on COVID-19?
   1. *Emergency services*
   2. *Guidance to accessing services at night*
   3. *SRH services*
   4. *People from remote areas – [how different was it from people in urban areas]*
5. What is the government is doing to ensure that SRHR remains a priority during the COVID-19 pandemic?
6. What strategies did the government take to improve health care services since the pandemic?
   1. *To increase accessibility and availability of healthcare services?*
   2. *To increase affordability of healthcare services?*
   3. *To guarantee an enabling working environment for healthcare workers?*
7. What are some of the impacts of the actions by government in response to COVID-19?

a. Which strategies were effective?

b. Which strategies were ineffective?

1. What are some of the barriers to accessing SRH services during the COVID-19 pandemic?
   1. What has the government done to address the barriers to accessing SRH services?
   2. What challenges is the government facing to ensure that SRH services are available?
2. Are there interventions targeting historically marginalized groups during the COVID-19 pandemic
   1. *Young people*
   2. *Sexual and gender minorities*
   3. *People with disabilities (PLWD),*
   4. *Indigenous communities*
3. What is the government doing to ensure sustainability of PPEs supplies for Health Care Providers?
4. Are there any emergency powers invoked by your government in response to COVID-19? Are there any limitations? Yes/ No Or controls over these powers?
5. Is there an existing mechanism to address violations of the right to health during this pandemic?
6. Is the judiciary in your country well prepared to deal with any human rights violations happening during the COVID-19 pandemic? If yes kindly, explain your answer
7. What do you want the donors/NGOs/CSOs community to do to support the response to COVID-19 pandemic?
8. Are there any safety nets for Healthcare?
