# Supplementary material for: Impacts of COVID-19 on reproductive health service provision, access, and utilization in Ethiopia: Results from a qualitative study with service users, providers, and stakeholders
Source: PLOS Glob Public Health. 2023 Mar 23;3(3):e0001735. doi: 10.1371/journal.pgph.0001735 (PMC10035746; doi:10.1371/journal.pgph.0001735)
Supplement: S5 Text — (DOCX) [file pgph.0001735.s005.docx]

**De-identified data:** *Impacts of COVID-19 on reproductive health service provision, access, and utilization in Ethiopia: Results from a qualitative study with service users, providers, and stakeholders*

| **Theme 1: Impacts of COVID on SRH access and service utilization** | | |
| --- | --- | --- |
| **Sub-themes** | **Source** | **Quotes** |
| ***Stakeholders and providers*** | | |
| Decreased service utilization | Policy maker | During COVID-19 pandemic, the trends of service utilization has declined. Reports on service use indicated us declined trend. We discussed among ourselves and started to conduct supportive supervisions to support regional teams, mobilize SRH resources from partners and revitalize SRH services |
| Reduced service provision; Reproductive health implications | Policy maker | Conference among pregnant women had been conducted up to 3 times per month at the waiting room in each health facility, but due to COVID-19 pandemic, it is discontinued for 9 months, and home delivery had increased as a result |
| Transportation costs | NGO/CSO representative | Transportation cost doubled during COVID-19 pandemic because of COVID-19 caused restrictions. To maintain social distancing, transportation services restricted to use one third of their seats |
| Changes to contraceptive counseling | Provider | We also counseled mothers to shift from short to long-acting FP service to reduce the risk of COVID-19 infection for frequently visiting the health facilities |
| Reduced service provision | Provider | NCD client’s appointment is extended to every 3 months from every month |
| Reduced service provision | Provider | We reduced the frequency of visiting health facility for lactating and pregnant mothers to minimize their risk of COVID-19 infection |
| Shift to virtual service provision | NGO/CSO representative | Providers started to give virtual support and counseling for abortion services |
| Consequences of COVID mitigation measures | Provider | The cost of face mask was one of the main complaints of clients. As a result, patients do not want to wear face mask. Clients need fast service; they do not want to stay in health facility due to fear of COVID-19 transmission in health facility |
| Consequences of COVID mitigation measures | Provider | Most clients complain about COVID-19 prevention strategies. Pregnant women do not want to wear face mask. On the other hand, providers are not willing to provide service those who are not wearing face masks on no mask no service rule |
| Fear of infection | Provider | In fear of COVID-19 infection, HIV/AIDs patients, FP, ANC, and PNC clients, had great fear to visit health facilities |
| Decreased service utilization | NGO/CSO representative | As a result, flow of such patients has significantly reduced as evidenced from our periodic reports |
| SRH outcomes; Decreased service utilization | Policy maker | Consequences of COVID-19 pandemic continues to manifest; still ANC and PNC service uptake has not yet been improved. Consequently, home delivery and maternal mortality expected to increase in the future. Except ANC services, all other SRH services has been discontinued during the initial phase of COVID-19 period. |
| SRH outcomes; adolescents | Policy maker | Due to COVID-19, most schools were closed –early pregnancy and teenage marriage increased, and GBV has increased |
| Increased GBV | NGO/CSO representative | Rape and GBV were some of the events aggravated during COVID-19 pandemic. We noticed that GBV is among the negative health outcomes observed during COVID-19 period |
| Increased GBV; SRH outcomes | NGO/CSO representative | The incidence of HIV raised in conjunction with GBV cases |
| SRH outcomes; adolescents | NGO/CSO representative | I noticed a higher incidence of teenage pregnancy during COVID-19 period |
| Abortion incidence | NGO/CSO representative | We have observed that incidence of unsafe abortion increased in the era of COVID-19 pandemic |
| SRH outcomes | NGO/CSO representative | Population expected to increase due to low use of family planning and high frequency of sexual intercourse during stay at home |
| SRH outcomes; adolescents | NGO/CSO representative | We observed different impacts of COVID-19 on the wellbeing of the community. Community has experienced such negative health outcomes as psychological disorder, family conflict, social media addiction, substance abuse, obesity and overweigh. These problems are more observed on students |
| SRH outcomes; adolescents | NGO/CSO representative | Our primary clients are students. Most universities closed during COVID period and students severely affected by the event. Some students did not want to join with their family, they started prostitution, worked in café and restaurants and become daily laborers to cope up their economic burden. Some students exposed for teenage pregnancy and unwanted pregnancy. This leads to abortion too |
| ***Health service clients in Addis Ababa*** | | |
| Changes to service provision | 25-year-old, married woman | R. Most people do not keep their distance from others. Otherwise, I don’t see any difference in service provision before and after the COVID-19 pandemic except applying the protective measures. The health worker provided me with adequate information about contraceptive methods. And he was caring and friendly in providing the service. |
| Fear of infection; barriers to facility access | 25-year-old, married woman | R.. Most women are not visiting health facilities in fear of COVID-19 infection. The SRH information and services should be provided door to door through health extension workers/ community health workers |
| Facility access | 21-year-old married woman | R. I had not encountered any challenge to visit the facility-The facility is close to my house and there was no curfew/restrictions or lock down because of COVID-19 pandemic. |
| Facility access; mitigation measures | 21-year-old married woman | No challenge and not affected at all, except applying COVID-19 infection protective measures. I had tried to reduce the of contracting COVID-19 infection by applying the protective measures. |
| Service provision; positive facility experience | 21-year-old married woman | I don’t see any difference in service provision before and after the COVID-19 pandemic except applying the protective measures. There were many service seekers around the registration (card) room. The health provider was good at understanding my concerns, providing information and service, he treated me in a good manner. The health facility is clean and attractive. |
| Barriers to facility access | 30-year-old married woman | R. There were many service seekers around the registration (card) room. Most people do not keep their distance from others. The health provider did not provide the service timely-I had to wait long time to get the service. Also, they do not provide full information about contraceptive methods. The service room is narrow in space and not comfortable. |
| Facility access; positive facility experience | 26-year-old married woman | R. I did get the service I wanted. No challenge. The counseling was good, Health Care Providers are friendly and compassionate. The environment is good: Hospital cleanness is acceptable. |
| Transportation barriers | 33-year-old married woman | R. Although the facility is not far from my home, there is transportation problem here in Addis Ababa, especially in this difficult time, I have to wait long time to get a taxi and visit this facility. Once I get here, I did not encounter any challenges to get the service as I know the service points before. I was a little busy to come on time and receive the contraceptive service. |
| Positive facility experience; Facility access | 33-year-old married woman | R. The health care providers are taking protective measures to prevent COVID-19 infection, so they took a little longer time to serve a client. As a result, we should wait a bit longer time to get the service. Otherwise, I got the service I sought. The counseling was good, the health worker has provided me with adequate information. The service was private, friendly, and confidential. The environment is clean and conferrable. |
| Protective measures; facility experience | 42-year-old married woman | R. The service is better now, before COVID-19 pandemic all service seekers were registered in one room and there was long line to get the patient card and, now registration is made in the respective service points and no crowd of people. Of course, we should take protective measures such as social distancing, using face masks and sanitizer in order to get the service. Otherwise, the health care service is good, I have no complaints. The procedure and waiting space is good and clean. Providers and staff were friendly and caring, I don’t have any reservation to comment. |
| Impact on contraceptive decision-making; fear of infection | 42-year-old married woman | R. The COVID-19 pandemic did not affect my choice of SRH or contraceptive services. But I delayed to remove the implant, I couldn’t come on time because of fear of COVID-19 infection. At the start of COVID-19 pandemic, everything was scary and affected our movements, I stayed at home two months when the pandemic started and delayed the removal. |
| Facility access | 23-year-old, married woman | R. Because of COVID-19 pandemic, I didn’t face any challenge to get the service. There were no curfew/restrictions or lock down because of COVID-19 pandemic. There was many people around the registration (Card) room. The facility is close to where I live, it is situated within the walking distance. No problem from my husband. |
| Facility access | 23-year-old, married woman | R. I don’t see any difference except taking protective measures such as social distancing, using face masks and sanitizer. Waiting time is a bit long, I had to wait more than an hour to get patient card. The procedure and waiting space is good. Providers and staff were respectful and caring. |
| Facility access | 25-year-old married woman | R. I didn’t experience any challenge to visit the facility, there were no curfew/restrictions or lock down because of COVID-19 pandemic. My partner was also supportive. The facility is close to where I live, I Know it very well and there was no transportation problem. |
| Fear of infection | 31-year-old, married woman | R. During COVID-19 era I had a fear of COVID-19 infection and was hesitating to visit health facilities. Once I got here, there were many people around registration room, and it was intimidating. Otherwise, the service counseling was good, the health worker was friendly and respectful. |
| Mitigation measures; Facility experience | 31-year-old, married woman | R. The people in the facility do not keep social distancing, and they are negligent in taking protective measures-clients are not following directives to preventing COVID-19 pandemic. However, the health care Provider are taking the protecting measures, they use face mask and keep social distancing during counseling and use Alcohol, glove and face mask while doing the procedure. |
| Mitigation measures | 33-year-old, married woman | R. The only difference is taking protective measures such as social distancing, using face masks and sanitizer. The service is good, and the facility is acceptably clean. |
| Wait times | 31-year-old married woman | R. The facility is close to where I live but there was a delay in getting the service. Providers are not on job in time |
| Contraceptive counseling | 31-year-old married woman | R. Before the pandemic, I was not allowed to take Injectables from this facility, health works were pushing me to take implants and I have to go to private clinics and pay for the service of my choice. After the pandemic, today I have got the service I sought. The service is good except some time delay to get. The provider was friendly and caring during counseling and providing the service. |
| Community outreach | 31-year-old married woman | R. Government should expand access to SRH information and education to all through various means including door to door visit. |
| Positive facility experience | 22-year-old, married woman | R. I did not come across with any challenges. I am taking preventive measures to protect myself from COVID-19 infection. |
| Contraceptive counseling | 26-year-old married woman | R. Sometime some providers are pushing clients to prefer a specific method, especially LARC methods and on the other hand, some women have misconceptions towards LARC methods. So, there must be a strategy to reduce providers’ biasness and alleviate clints’ misconceptions. |
| Fear of infection | 40-year-old married woman | I had a fear of COVID-19 infection while I came to the facility. Since there are many service seekers in the facility, I lack confidence that I and my child might be infected by the virus. The facility is close to where I live and there is no restriction or curfew where I live. I had shortage of time to visit the facility as I was busy. |
| Fear of infection | 40-year-old married woman | R. The risk or fear of COVID-19 infection is the major change; you have to wear mask and protect yourself from the virus. The crowd around the registration (card) room was very intimidating, most service users do keep their distance from each other. Otherwise, the health providers were friendly and respectful, they treated me in a good manner. |
| Mitigation measures | 33-year-old married woman | R. I don’t see any difference in service provision before and after the COVID-19 pandemic except applying the protective measures. I have received the service I needed, there were no service interruption because of the pandemic. The health worker was good and provided me with adequate information about contraceptive methods. He was compassionate and friendly while providing the service. |
| Mitigation measures | 30-year-old married woman | R. I have observed that service seekers do not keep the social distancing in the compound they were near to each other, I think the facility must do something to implement all the protective rules properly. |
| SRH priority | 30-year-old married woman | R. COVID-19 shifts SRH priorities and some SRH issues such as HIV/AIDS, STI and abortion are being neglected, government should exert more effort to make all SRH related problems are of high priority. |
| Mitigation measures | 24-year-old married woman | R. After the pandemic, I have to wear face mask, keep social distancing and use sanitizer. Today, there were many people seeking the service and not keeping their social distancing among each other, I had to wait a considerable time to get the service. But in general, I’m satisfied by the service and I got the service I needed and the health facility is good in terms of cleanness. |
| Fear of infection | 36-year-old married woman | R. I had stopped visiting the facility for a while in fear of COVID-19 infection. |
|  |  |  |
| ***Health service clients in Amhara*** | | |
| Partner involvement; contraceptive choice | 35-year-old married woman | R I have some challenges. My husband does not support me to use FP. He needs to have child from me. Now I have one child from him, he allows me to use family planning method. Otherwise, I do not have other challenge. |
| Partner involvement; contraceptive choice | 35-year-old married woman | R. I delivered a baby during COVID and now I consulted with my husband to use FP. After we have a child, my husband allows me to use FP method. |
| Mitigation measures; Barriers to access | 35-year-old married woman | Sometime I unable to get face mask and providers do not give service without face mask. |
| Contraception use; SRH information | 35-year-old married woman | R Education is necessary, my friends do not need to use FP. The do not have detailed information. I hide my friends when I used FP. This is to keep my privacy. I afraid my friend if they know me to use FP. The community health development cadre do not teach us well because they do not receive per diem. |
| Transportation; Cost of FP; positive experience | 25-year-old married woman | R My home is near to this health facility and I do not have transport problem. My husband is willing for me to use FP. The cost of FP is free. I do not have any challenge to get FP service. |
| Transportation; Fear of infection; Supply shortage | 26-year-old married woman | R I referred to this hospital from other health center. I have difficulty to get transport. I couldn’t easily get transport. The cost of transport is high. I have information about this hospital to get abortion service. I have no difficulty to get the hospital. There is shortage of drugs in the hospital. There was four clients in a single room. We had fear of COVID contamination. |
| Fear of infection | 26-year-old married woman | R. I affected psychologically. I was stressed during my stay in the hospital due to fear of COVID infection |
| Mitigation measures | 26-year-old married woman | R. Before COVID service users can explain their history but during COVID there are communication barriers due to distance and face mask. I did not hear properly the sound of provider. |
| Fear of infection; mitigation measures | 26-year-old married woman | R. I had fear of COVID infection. I used face mask and sanitizer but social distancing was difficult for me. The room in the health facility is narrow. We shared materials. There was no soap to wash hand. |
| Mitigation measures | 26-year-old married woman | R. The health provider restricted the number of caregivers to avoid crowded. Providers and service users use face mask. Service users share materials which leads to COVID contamination. The providers appointed me for two months which is long. |
| Provider shortage; sanitation concerns | 26-year-old married woman | Quality of service need to be improved. There was shortage of providers and I recommend the health facility to add providers. Providers do not change glove and the facility should avail supplies to the provider. |
| Mitigation measures; Wait times | 30-year-old married woman | The health facility guard enforced me to use face mask, inform us to keep distance at card room and order to wash our hand. This takes time and I waited sometime to get the service. Providers offered me good service timely. |
| Mitigation measures | 30-year-old married woman | R. The health providers informed us to wear face mask, to wash our hand and keep our social distance. There is water and soap at the entrance. I saw many people who returned from the entrance due to lack of face mask. This may expose women for unwanted pregnancy due to lack of face mask. |
| Mitigation measures | 30-year-old married woman | R. For me, COVID does not have effect to use health service but for other people who have no face mask, it prevents from using the service in the health facility. The facility does not provide service without face mask. I have this information and came with face mask. |
| Geographic access barriers; SRH information | 30-year-old married woman | R. SRH and FP services should be available in health center and health post. Sometimes health posts closed in rural areas and service users returned without getting the service. I recommend the health posts need to be opened all the time. The concerned body shall provide education through mass media. For rural women information could be reached through conference. |
| Geographic access barriers; SRH information; Partner involvement | 22-year-old, single woman | R. I do not have time to visit health facility. The health facility is far from my residence. I do not have much information about the health facility to get abortion service. My boyfriend is not willing to visit this health facility. I would take FP service if the health facility is near and my partners encouraged me. Because of distance, lack of information and lack of willingness from my partner I exposed for unwanted pregnancy. This affects my health. |
| Barriers to access; positive experience | 22-year-old, single woman | R. During COVID providers were not willing to provide service. We can’t get providers on time. Now I obtained the service. I satisfied when the provider gave me the abortion service. I think the privacy is good. The provider is treating me friendly. I await sometime at card room, but the service is fast at abortion service delivery point. |
| Fear of infection; FP access | 22-year-old, single, student | R. During COVID I have fear to obtain FP at health facility. I was unable to use FP and exposed for unwanted pregnancy. |
| FP access; Mitigation measures | 30-year-old married woman | R. I am here to take FP. I used FP before and during COVID. There is a difference before and during COVID. During its initial stage of COVID pandemic, FP method was not available when I need it, providers said to us there is not PF. Now FP is available but still we can’t get it immediately a like that of before COVID. The process to get FP service now is long. Guards at the entrance push to use wash our hand and keep distance. I could not get FP without face mask. |
| Geographic access barriers; Transportation; Cost | 30-year-old married woman | R. My home is far from the health facility. I do not have time since I had children to take care of them. There was no transport – I paid double for transport. |
| FP access | 30-year-old married woman | R. COVID affected my FP uptake. I become pregnant due to lack of FP during COVID period. |
| FP access; Transportation costs | 30-year-old married woman | R. I took FP from private health facility. I have economic burden to pay for transport. I came on foot. |
| FP access | 30-year-old married woman | R. The health facility is clean. There was lack of FP availability. Providers restricted to provide for only some women. Immediately during COVID pandemic, I could not get FP. Now I am satisfied on the service I got. |
| FP access; Cost | 30-year-old married woman | R. I need three months injectable FP, but I could not get it during COIVD. So COVID affected my FP choice. Due to unavailability of injectables in this health facility I tried to get it from private clinic, I spend extra const to get it. |
| FP access; SRH service availability | 30-year-old married woman | R. I prefer to get FP in our near health post. If possible, government should avail FP in all health posts to easily access FP methods. This will prevent from extra cost for transport and time. Service discontinuation need to be improved. |
| Geographic barriers to access; Transportation | Missing demographic information | R. I have challenges to get the service. The health facility is far from my home. I live in rural area. Previously we do not have transport. Transport was closed during COVID. |
| Geographic barriers to access | Missing demographic information | R. I recommend, health posts near to our home. The location of health facilities is far from our home. We need FP services in our near health facility and we need education in school near to our home. |
| Geographic barriers to access; Transportation | 28-year-old married woman | R. During first phase of COVID pandemic, the health facility is far from my home. There is no transport facility in our locality. I traveled an hour from my home to this health facility. |
| Geographic barriers to access; Transportation | 28-year-old married woman | R. The distance does not affect my health and choice to use services, except I tired while I travel long distance from my home to this health facility … I travel on foot. |
| Mitigation measures; Fear of infection | 28-year-old married woman | R. I use face mask, sanitizers, wash hand and keep social distance. Some service users do not use face mask and I had fear. Personally, I do not have challenge to use these COVID prevention methods. |
| Fear of infection; FP use | 28-year-old married woman | R. COVID has impact on my choice of SRH services. I had fear of COVID during the first phase of its pandemic and I interrupted to use FP service |
| Transportation; Cost; Geographic barriers to access | 22-year-old, single woman | R I had transport problem – the cost for transport during COVID is increased. Transport was also not available. The health facility is far from my home. Most taxi drivers need contract reasoning that there is shortage of fuel. I am student and I do not have money/income. The service provision time is not suitable for students. We are relatively free at lunch and evening but the service is not available during lunch and evening time. We can’t also get the service during the weekend time. |
| Service availability; transportation | 22-year-old, single woman | R. Most of my problems to get the service were related with transport and time mismatch to get service. Providers do not available in weekend time, I went to private health facility. It makes me extra expensive- unnecessary cost. This affects my psychology and I was stressed. |
| Cost; Transportation | 22-year-old, single woman | R. I enforced my families to send money to get this service. I used the money to purchase drugs and used for transport cost. While I unable to get the service during weekend time, I got the service from private clinic and use drugs from pharmacy. |
| Mitigation measures; Stigma | 22-year-old, single woman | R. Personally, I used face mask and hand washing but social distance is challenging –particularly during transport and market place. As I saw, providers and clients do not use face mask. They are ignorant to use face mask. It needs strong monitoring system in this health facility to use face mask. Specially in Gondar town, most people do not use face mask. The big problem is social distancing. When you use face mask – some people perceive you had COVID. People had fear of stigma when you use face mask. The awareness is created in the community but most people are ignorant unless they experienced with COVID. |
| Mitigation measures | 22-year-old, single woman | R. The health facility took COVID prevention measures, for example; they enforce to use face mask at the entrance. There is hand washing facility but not functional and do not have soap and water. There is no controlling system for face mask use in health facility. There is temperature measurement at main gate. Providers give due attention to protect themselves. |
| Service availability; Positive experience | 32-year-old married woman | R. I have no problem to use service. The service is open every time. I do not have time problem to use the service. I have no difficulty to get information about the service. My residence is near to this hospital. My husband is supportive to use ANC service. |
| Fear of infection; Mitigation measures | 32-year-old married woman | R. Almost in a month we do not visit health facility. Schools were closed. We had fear due to COVID and we put papers and materials out of home. We use sanitizer and wash our hands. We discontinued our social with other people. We stay at home. W use face mask purchasing from Bahir Dar. Our choice for COVID prevention was to stay at home. |
| Fear of infection | 32-year-old married woman | R. COVID has impact on SRH service use. Most women do not come to health facility due to fear of COVID. People had fear of quarantine- isolation, fear of stigmatization from their families due to COVID infection. Even for me I did not come into health facility during its first phase of COVID pandemic. |
| Geographic disparities in access | 32-year-old married woman | R. The urban community have information to prevent COVID and use SRH services. But rural community do not have the chance to get information. Providers should educate rural community and health post workers through outreach program. Health facility guards should get education to manage service users. Sometime we may not get providers on the time when we need, so providers should be available every time. |
| Geographic disparities in access; Transportation | 20-year-old married woman | R. I lived in rural village and I have difficulty to get transport facility. My home is very far from this health facility. I have information FP and abortion services are available in this health facility. Providers educated us. My husband is willing to use this service. |
| Geographic disparities in access; Transportation | 20-year-old married woman | R. Transport for rural women is big challenge and the health facility need to arrange transport facilities for women who have severe cases. It would be good if there is a health center to be constructed near to our residence like that of health post. |
| Abortion experience | 20-year-old married woman | [Post-abortion] R. I had fear since I had heavy bleeding. I call to the HEW and she send me ambulance. |
| Positive experience | 25-year-old single woman | . The hospital is not far. I do not use any service before COVID. I do not have challenges; every step was good. I got all the service I want. I satisfied for the service. I was happy while my abortion is terminated. Providers are friendly. The privacy is kept. Abortion room is separate and it is clean. |
| Geographic disparities in access; Transportation; Abortion experience | 25-year-old single woman | R. There is fear and stigma for safe abortion. Women need special education and counselling to minimize such challenges. For rural women they need road transport facility. Priority need to be given to old people to get the service. |
| Transportation; Mitigation measures | 23-year-old married woman | R. There was transport shortage and increase cost of transport during COVID. Movement form place to place and transport was restricted. Otherwise, I do not have challenges to visit health facility.  COVID affected me to use FP and other health services from health center due to lack of transport. |
| Mitigation measures | 23-year-old married woman | R. I stay at home. I did not use health services during the first phase of COVID pandemic. |
| Transportation; SRH impacts; FP access | 23-year-old married woman | R. COVID affected our health through lack of transport to receive FP. Furthermore, during COVID, demand of sexual intercourse from my husband increased. Unplanned pregnancy occurred. |
| Transportation; Cost; Service availability | 23-year-old married woman | R. In order to get SRH service it is better to increase transport facilities, and balance the cost of transport. It is better to increase the number of providers during COVID time to reduce their work load. Students interrupted from school and their family pushed them to marry. Providers need to educate the community to prevent early marriage. |
| FP access; Fear of infection; Mitigation measures | 23-year-old married woman | R. I am here to use FP method, specially injectables for three months. I used this method before now. I have difficulty to use the service due to fear of COVID. Before COVID, the service was good. I do not wait long time to get the service except at card room. During COVID, providers enforced us to use face mask. It is difficult to get face mask if I have no money. |
| Transportation; Cost; FP use; Partner involvement | 23-year-old married woman | R . I have transport problem – the cost for transport is high. The cost of transport is high and even if you get it is crowded. My husband does not allow me to use FP. He does not know when I come to here for FP method. The health center is far from my home |
| Transportation; Cost; Geographic disparities to access | 23-year-old married woman | R. COVID does not affect my heath. But it affects my life through transport cost. I prepare money for transport. I came on foot thought it is time taking. I must use face mask when I go to health facility. When I come to health facility I do not want to inform to my husband. It a secret. |
| SRH access; Mitigation measures; FP access; Transportation | 23-year-old married woman | R. Before COVID I do not wait long time except at card room. During COVID, there are a lot of challenges – providers and health facility guards enforced us to keep social distance, to have face mask and wash our hands. It is difficult to enter into the health facility and get service without face mask. Waiting time is too long. Previously I used injectable FP method but no I used FP for three years. This is because I have difficulty to get transport. |
| Mitigation measures; Cost | 23-year-old married woman | The other challenge is that the cost of face mask is high and the size of the face mask could not fit with my face. My relation with my neighbors is discontinued due to fear of COVID. |
| Fear of infection; FP use; SRH outcomes | 23-year-old married woman | R. Due to fear of COVID and transport problem I discontinued to use FP. During COVID I faced unwanted pregnancy. I deliver a child without my plan. COVID affected my FP service use and had great impact on my life. |
| FP use; Educational efforts | 23-year-old married woman | R. Providers should to educate us when we come to health facility. I need detailed information about the advantage and disadvantage of FP methods. Women do not accept new family planning methods – most women interested to use a three-month FP. This exposed to the cost for transport to visit health facility in every three months. If providers educate us the advantage and disadvantage of FP, we can use a three-year FP method. I will inform to my friends to use the three-year FP method if it is comfortable for me. |
| Transportation; Mitigation measures | 35-year-old married woman | R. There is transport problem. I have no problem from my husband to use FP methods. We were confused when providers informed us to use face mask. It is terrible for us to use service but now we adapted. I have pain while I use FP spacing. |
| Transportation | 35-year-old married woman | R. I come to hear on foot. I learned about COVID to reduce my stress. |
| Fear of infection; Communication; Mitigation measures | 35-year-old married woman | . During COVID we have fear. Providers do not communicate closely with us. They seat far from us and we do not hear their sound. Providers enforced us to wear face mask. This health facility is clean and providers are welcoming but I need to obtain service in the health post that is near to my home. I visit this health facility when I come to purchase something from this town. |
| Youth; Fear of infection; Service availability | 35-year-old married woman | R. COVID has many impacts. Students discontinued from university and come to home. COVID created fear to use health facilities. Health posts usually closed during COVID due to COVID fear. |
| Geographic disparities in access; Service availability | 35-year-old married woman | R. Health posts need to be opened. Usually, health posts closed. I need services near to my home. I need COVID vaccine like that of urban people. |
| Service availability; Fear of infection | 32-year-old divorced woman | R . My residence is near to this health facility. Sometimes, the heath facility closed during COVID. I had fear to get service due to COVID infection. There are misconceptions about COVID and fear developed in the community. Sometimes providers ordered us to purchase from private pharmacy. |
| Service availability; Mitigation measures | 32-year-old divorced woman | R. Service provision has difference during and before COVID. Before COVID I came with my friends but during COVID I can’t come with friends. I have to wear face mask to obtain service. Providers and admin staffs are cooperative but the service is time taking. I await long time to get the service. |
| FP availability; Educational efforts | 32-year-old divorced woman | R. The health facility should avail all types of FP methods. DEPO injectable FP not available in the health facility and the health facility must think to avail in this health facility. Health providers need to make face service starting from entrance, card room and FP unit. Providers need to give detail information and create awareness about different types of FP methods. I would be happy if the provider tells me in detail about the side effect and benefits of each FP methods. |
| FP availability; Positive experience | 27-year-old married woman | FP service provision has no difference during and before COVID. I have no any challenge to get FP service from this health facility. |
| FP availability; Mitigation measures | 27-year-old married woman | R. In order to get FP service I must wear face mask, hand washing and keep distance from other people. There is a challenge. Clients wear face mask to enter in the health facility. We wear face mask for the purpose to obtain service. Women give their face mask to other people after return from health facility. |
| Abortion experience | 22-year-old divorced woman | R. I did not disclose my pregnancy and termination to my family members. I informed other reason to my families. I informed them to obtain injection for other disease. |
| Transportation; Family involvement | 22-year-old divorced woman | R I am will to visit this health facility in the future. Lack of time and family’s willingness were my challenges to come to obtain this service. The health facility is not far from my home. I do not have difficulty to come to this health facility. My families do not know my pregnancy. If they know they are not willing to terminate my pregnancy. |
| Abortion experience; Fear of infection | 22-year-old single woman | R. I come to terminate my unintended pregnancy. It is my first visit in this health facility. After my termination I will take FP services. I did not use FP during COVID time. I had fear to go to health facility because of COVID. |
| FP availability; FP use; Unwanted pregnancy; Abortion experience; Positive experience | 22-year-old single woman | R. Before COVID I took what I need. I took DEPO FP type for three months. It was available. But during COVID I could not get DEPO in the health post and the provider gave me oral pills for one month. I forgot to take pills and now I faced unwanted pregnancy. Now I received the service what I wanted. But my abortion procedure is not finalized yet. I am satisfied on the service up to now. There are good things in this health facility – providers are friendly. |
| FP availability | 22-year-old single woman | R COVID is not affecting my service choice. But some of my friends informed me implanon and LUP has disadvantage and it was not my choice. |
| Transportation; Fear of infection | 26-year-old single woman | R I do not have problem related with transport. I had fear to get service. I missed my work. I did not get detailed information. |
| FP availability; Fear of infection | 26-year-old single woman | R COVID does not have any impact to choose FP. But I had fear and stress to use the service. |

| **Theme 2: Impacts on the health care system and service provision** | | |
| --- | --- | --- |
| **Sub-theme** | **Source** | **Quotes** |
| Service prioritization; Service availability | NGO/CSO representative | During lockdown, health facilities were not providing health care services other than COVID-19 prevention and treatment. The focus of health facilities was towards COVID-19 prevention and treatment |
| Mitigation measures; Transportation; Service availability | NGO/CSO representative | The government response to COVID-19 pandemic, particularly the lockdown strategy had significant impact on SRH service provision, most health facilities were closed, and transport service were restricted, health care providers were also restricted to go to the health facilities The health system was collapsed and coordination was so poor, the linkage between health facilities-health posts (HP) and health center (HC) and hospitals was severely affected. HEWs used the lockdown strategy as a good opportunity to stay at home and do their own things |
| Mitigation measures; PPE availability | Client | Face mask is mandatory to get FP service in this health facility. However, there is no water and soap at the entrance of the health facility |
| Mitigation measures | Client | At the entrance of the health facility, they informed us to wear face mask and keep social distance. At the entrance there is water and soap, and we have to wash our hands. I always wear face mask when I go outside of my home |
| Mitigation measures | Client | The health facility posted a message at the entrance to wear face mask, keep social distance and wash hands with water and soap. The messages are good to aware health care seekers; however, it is difficult to keep social distance at card room |
| Mitigation measures | Client | At the entrance of the health facility, they measure body temperature, control us to wash our hand and wear face mask |
| Mitigation measures | Client | The health facilities informed us to use face mask at the entrance. However, there is no controlling system for face mask use in health facility |
| Mitigation measures; Service provision adaptations | Policy maker | Health facilities were providing SRH services with no mask – no service rule, they also adjusted seating service provision arrangements to prevent COVID-19 infection. Service appointments were also extended, ART drug appointment extended to 6 months, ANC attendance changed from 4 visits. We extended population catchment areas for COVID-19 prevention and management, and it was our day-to-day priority tasks |
| Service availability; FP availability; Commodity shortages | Provider | RH services like FP and ARV are exempted for free however they are not available at all times for many reasons, there is service interruption, particularly because of shortage of commodities, supplies and equipment |
| Commodity shortages; FP availability; Funding adaptations | Provider | We have shortage of drugs and supplies due to COVID-19 disruption and due to large number of displaced people (from Benishangul) seeking the service. In my Woreda, you can’t get FP commodities in most of the health facilities except the injectable. NGOs … had stopped their support during COVID-19 pandemic and … is not providing essentials commodities and supplies timely for our health facilities |
| Commodity shortages | Provider | There were no sufficient drugs and commodities during COVID-19, particularly, medical drugs, surgical glove, and ART drugs. Shortage of essential commodities and supplies was the commonest challenge to provide SRH services |
| Commodity shortages | Provider | commodities and supplies like catheter, antipain medications, IV medication, and PPE materials are reduced in availability and was almost zero level in some facilities for some time during COVID-19 pandemic. MA drugs and FP commodities becomes was frequently interrupted. The main reason is priority shifting to COVID-19 pandemic prevention and shortage of budget |
| Provider stigma | Provider | Health providers’ social interaction and engagement was severely affected by COVID-19 pandemic. I personally was unable to get rental house because of the community stigma. They perceived me as the carrier of COVID |
| Provider stigma | Provider | I personally negatively affected by COVID-19 pandemic. I also had anxiety/fear to join with my family while to return to home. I had difficulty to get rental house |
| Provider workload | NGO/CSO representative | COVID-19 increased workload among providers including me. We are engaged in COVID-19 prevention taskforce, and it has created additional burden to our work |
| Provider workload | Provider | Additional tasks associated with COVID-19 vaccination is becoming another burden for health care providers and it affects SRH service provision. COVID-19 increased workload among us, we should stretch ourselves to cover the work of staffs who died of and infected with COVID-19 virus, there was no immediate replacement for these providers |
| Fear of infection; Provider illness | Policy maker | Initially, health service providers and office workers had higher anxiety and fear of COVID-19 infection. Many providers and staffs were infected by COVID-19 virus and some of them died. After the orientation on infection prevention, infection rate has significantly reduced, things seem to return normal |

| **Theme 3: Adaptations made by the health system, providers, NGOs and communities** | | |
| --- | --- | --- |
| **Sub-themes** | **Source** | **Quotes** |
| Educational efforts | NGO/CSO representative | We had difficulty to create demand for SRH services in the community, since gathering of people was restricted, but we used our promoters to create demand for service through home-to-home visits by applying COVID-19 infection prevention strategies |
| Educational efforts; Media | NGO/CSO representative | The government developed different strategies to engage local communities in COVID-19 response initiatives and COVID-19 pandemic awareness activities. Various information had well been announced through health education at HFs and mainstream medias |
| Educational efforts | NGO/CSO representative | We established free message code center 844 with the objective of providing information about COVID-19 pandemic. The center is free and our clients get information for and receive counselling services if they needed. We included COVID-19 issues in the discussion and community consultation |
| Educational efforts; Media | Policy maker | We used mass medias to aware the community about covid-19 pandemic |
| Educational efforts; Media | NGO/CSO representative | We used social media to inform the community from where to get COVID-19 treatment service and for education and awareness creation |
| Mitigation measures; Service adjustments | Policy maker | We tried to develop initiatives to generate fund for PPE support. As a result, we were able to support the health system with face masks, sanitizer, and soap. We also engaged in distributing PPE materials from high resource districts to low resource districts. Some health facilities were good at preventing COVID-19 infection by using PPE effectively and efficiently |
| Mitigation measures; Service adjustments | NGO/CSO representative | As part of mitigating COVID-19 response, currently we are providing portable ultrasound and PPE materials for health facilities. The PPE supplies include whole cover suite, face mask, water cleaner and dispenser materials. Our support was mainly for public health facilities |
| Mitigation measures; Service adjustments | Policy maker | Health facilities were providing SRH services with no mask – no service rule, they also adjusted seating service provision arrangements to prevent COVID-19 infection. Service appointments were also extended, ART drug appointment extended to 6 months, ANC attendance changed from 4 visits. We extended population catchment areas for COVID-19 prevention and management, and it was our day-to-day priority tasks |
| Guidelines; Prioritization | NGO/CSO representative | Many SRH guidelines and documents have been prepared in the context of COVID-19 pandemic. These include non-COVID essential health services, RMNCAYHN self-care interventions technical guideline, safe motherhood, and COVID-19 response documents. Essential health service guideline developed to maintain SRH services. After the implementation of essential service guidelines, SRH become among priority services in the context of COVID-19 pandemic and SRH service provision issues started to diminish, though providers did not have uniform understanding on the guideline |
| Service integration | NGO/CSO representative | We did not recommend COVID-19 related interventions as a stand-alone initiative rather it should be integrated with other health and non-health programing |
| Service integration | Provider | We included COVID-19 awareness creation session as part of the morning health education |
| Service integration | NGO/CSO representative | We motivated and engaged health work forces to integrate SRH activities with COVID-19 prevention and treatment activities |
| Adaptations to programs | Policy maker | RMNCH directorate conducted various consultative meetings with key partners to increase partners support and engagement in SRH program |
| Adaptations to programs | Policy maker | We encouraged regions through supportive supervisions to identify challenges and narrow gaps; fulfil both human and material and provide technical supports, this will help to maintain SRH services during the pandemic |
| SRH budget adaptations | NGO/CSO representative | We replanned our SRH project budget to purchase PPE materials since we did not have budget for COVID-19 prevention. Funders have also started to shift their budget towards COVID-19 prevention. COVID-19 pandemic has also become an opportunity for funding, some donors had supported the COVID-19 relief and response strategies |
| Budget adaptations | NGO/CSO representative | There was no pre-planned budget for emergency responses such as COVID-19 and most of the COVID-19 response activities utilized from up to 30% of SRH and other health program budget, |
| Budget adaptations; NGO support | NGO/CSO representative | For example, we supported PPE for health facilities- HFs by shifting budget from SRH. Zonal health departments have also requested us to support their emergency preparedness plan |
| Sanitation and hygiene improvements | Provider | Client flow from communicable disease reduced at OPD. This may be due to increase in personal hygiene or sanitation. Hand washing practice improved due to one of the COVID-19 prevention strategies and as a result communicable disease such as diarrhea and common cold reduced |
| Sanitation and hygiene improvements | Provider | Before COVID, there was no handwashing facilities, now we had water tanker to practice personal hygiene and sanitation |
| Sanitation and hygiene improvements | Provider | COVID-19 pandemic had negative and positive impacts, one of the positive impacts is that communicable diseases reduced due to good hygiene practice |
| Technology challenges | NGO/CSO representative | We attained global meetings and conferences through online systems, however, we were sometimes challenged by connectivity problem or when the internet become down or unreliable |
| Technology; Service delivery; Trainings | NGO/CSO representative | We started to develop online platforms to reach and connect with trained providers. We also have started to initiate and introduce telemedicine in some big cities. Some staffs work at home using telephone and online communication means. In person trainings has been reduced from our plan and we have started to conduct only theoretical trainings through online system |
| Technology; office adaptations | NGO/CSO representative | We learned to adapt new normal by using various communication and working systems |
| Technology; office adaptations | NGO/CSO representative | Most organization had adapted work at home strategy and use technologies including telephone conversation, e-mail and zoom meetings to facilitate connection and team works |
